# Short-Term Outcome of Early Primary Total Knee Arthroplasty for Fractures Around the Knee in the Elderly Population: The Experience of a Secondary Healthcare Centre in Malaysia

**DOI:** 10.21315/mjms2020.27.4.6

**Published:** 2020-08-19

**Authors:** Ng Bing Wui, Muhammad Afiq Ahmad Anuar, Abdul Muttalib Abdul Wahid

**Affiliations:** Orthopaedic Department, Hospital Segamat, Ministry of Health Malaysia, Johor, Malaysia

**Keywords:** total knee replacement, elderly, knee osteoarthritis, fracture around the knee (supracondylar and tibial plateau fracture)

## Abstract

**Background:**

The management of fractures around the knee in the elderly population can be challenging due to the complexity of the patients and the fracture characteristics. In this study, we aimed to investigate the short-term outcome of elderly patients who had fractures around the knee and who were treated with primary total knee arthroplasty. The study included patients who were at least 70 years old with poor bone quality and who presented with a fracture around the knee that would be difficult to treat with open reduction and internal fixation (ORIF) as well as patients who were at least 55 years old presenting with severe concomitant knee osteoarthritis.

**Methods:**

This is a cross-sectional study in which all the elderly patients who underwent early primary total knee replacement due to trauma around the knee at the Segamat Hospital between January 2015 and June 2019 were identified. Data were collected from clinical and operative notes. The clinical outcomes of these patients were evaluated by the range of motion of the knee and the Knee Society Score (KSS).

**Results:**

Ten patients were identified to have undergone this procedure. Six patients sustained supracondylar femur fractures, two patients had tibial plateau fractures and two patients had concurrent supracondylar femur and tibial plateau fractures. The mean follow-up duration was 22.3 ± 13.9 months, the mean knee score was 87.7 ± 10.0 and the mean functional knee score was 56 ± 41.9.

**Conclusion:**

In this cohort, good short-term outcomes close to pre-fracture condition was noted in patients who did not suffer from any complications during the post-operative period. Two patients who had surgical site infection had lower functional knee scores. Another two patients with lower knee scores experienced surgical site infection of the distal tibia and contralateral fixed flexion deformity of the knee. Early primary total knee replacement remains a viable option in treating fractures around the knee in the elderly. Infection, which in this study affected 20% of the patients, is the main deterring factor in performing this procedure.

## Introduction

Fractures around the knee are traditionally treated with open reduction and internal fixation (ORIF) or external fixation and, eventually, total knee arthroplasty (1, 2). However, treatment of these fractures in the elderly population could be challenging for the following reasons. First, the osteoporotic bones of older patients can make manipulation, reduction and fixation difficult (3, 4, 5). Second, excessive soft tissue stripping could cause bone necrosis, resulting in delayed union or non-union (1). Third, delayed weight bearing could cause increased risk of secondary fractures due to falls and increased immobility related to complications (3). Fourth, intraarticular fixation on a pre-existing damaged osteoarthritic joint is related to poor long-term outcomes (3). Fifth, performing total knee arthroplasty on a patient with compromised soft tissue condition post-ORIF is associated with an increased risk of repeated intervention due to scarring, patella mal-tracking, difficulty in soft tissue balancing and the need to address axial malalignment (6). Finally, repetitive surgery and rehabilitation on already immunocompromised elderly patients can create additional physiological and physical stress, increase the risk of infection and compromise soft tissue condition, thus further worsening the general outcome. ‘One best surgery’ is often the choice for managing patients with multiple comorbidities due to complexity of health problems. A similar concept has long been applied to hip fractures, where total hip arthroplasty is no longer uncommon in the management of femoral neck fractures in the elderly and has shown good outcomes. Early primary total knee replacement could potentially bypass all the mentioned difficulties. In this paper, we report on the short-term outcomes of our patients who underwent early primary total knee arthroplasty between January 2015 and June 2019 at our centre after sustaining a fracture around the knee.

## Methods

This is a cross-sectional study. All patients who sustained fractures around the knee and underwent early primary total knee replacements at the Orthopaedic Department of Segamat Hospital between January 2015 and June 2019 were included.

Primary total knee replacement is indicated for patients who are at least 70 years old with poor bone quality and who presented with a fracture around the knee that would be difficult to treat with ORIF and for patients who are at least 55 years old presenting with severe concomitant knee osteoarthritis (7). The clinical presence of knee pain due to osteoarthritis prior to trauma is taken into consideration as one of the factors when a patient is indicated for total knee arthroplasty after trauma. Pre-operative fractures were classified using the Müller Arbeitsgemeinschaft für Osteosynthesefragen (AO) classification. The degree of osteoarthritis and osteoporosis was evaluated via radiograph. Pre-operative and post-operative evaluations were obtained from medical files and radiographs, and data for patients was collected from individual clinic notes, patient case notes, operating theatre (OT) registries and ward registries. Operating times and implants used were retrieved from the OT notes. Exclusion criteria included patients who underwent total knee replacement more than three weeks after the injury and patients who underwent total knee replacement due to post-traumatic osteoarthritis.

Patients who met the inclusion and exclusion criteria were identified by reviewing clinical notes and the OT registry. Consent was obtained by the investigator or co-investigator of this study during patients’ visits to the clinic. A clinical evaluation and survey was conducted during patients’ follow-up by using the Knee Society Score (KSS) survey form (8). Fracture healing, complications and signs of loosening were registered.

### Statistical Analysis

All data collection was kept anonymous to maintain confidentiality. Data collected were analysed using SPSS version 23.0. Demography is described in numbers and percentages for categorical data and continuous data is described in mean and percentages. The significance between each confounders and outcome score was analysed using an independent *t*-test, Mann-Whitney test and Fisher’s exact test.

### Operative Technique

All surgeries were performed by experienced surgeons and assistants. The standard pre-operative anticoagulant and antibiotic prophylaxis were used. The surgery was performed under combined spinal-epidural anaesthesia. A midline longitudinal skin incision and medial parapatellar approach was used to perform the surgery. Fracture fragments were reduced using pointed reduction clamps, cerclage wires or K-wires before standard total knee arthroplasty cuts were carried out. Any bone stock deficiency was covered with cementation. Both femoral and tibial components with or without a stem were cemented into place. Post-operatively, no splints or orthoses were used, and patients were allowed to bear weight depending on the comminution of the fracture pattern and the presence of other concomitant fractures. A standard physiotherapy regime was employed for patients who received primary total knee replacement for osteoarthritis. Patients who could not bear weight used crutches for 2–3 months.

### Assessment Tool

The Knee Society Score is an assessment tool that rates knee function. With regards to knee assessment, three main parameters were considered, namely, pain, stability and range of motion. The maximum knee rating score was 100 points. Functional assessment considered walking distance and stair climbing and deducted points if a patient used walking aids. The maximum functional score was also 100. Each score is presented separately. The Knee Society Score assessment was done regularly in clinic during patient follow-ups. Approximately 10 min was needed for the surveyor to complete each patient’s clinical evaluation and survey.

## Results

### Demographic Characteristics

**Figure 1:**
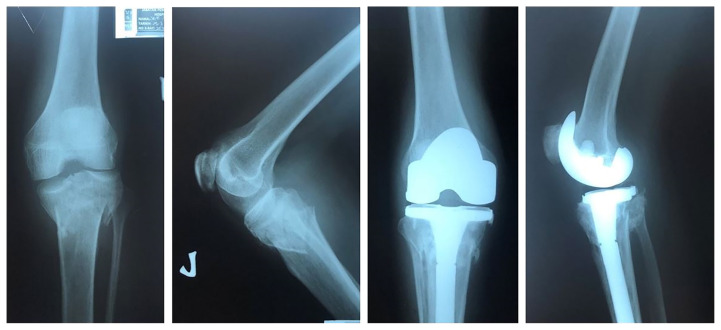
Complex proximal tibia fracture with pre-existing osteoarthritis changes managed with total knee arthroplasty

**Figure 2:**
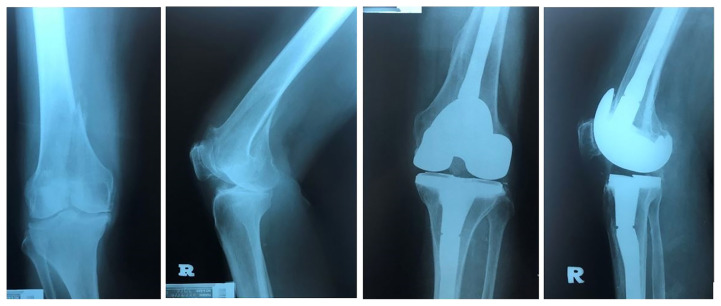
Intra-articular supracondylar femur fracture with pre-existing osteoarthritis changes and osteoporosis

A review of the data showed that 10 patients had undergone total knee arthroplasty within three weeks from the day of trauma. Due to the small sample size, inferences should be made with caution. Five of the patients were male and five were female with a mean age of 64 years and an age range of 55–76 years. Six patients sustained intra-articular supracondylar femoral fracture, two patients sustained complex intra-articular proximal tibia fracture (Figure 1) and two patients sustained both intra-articular distal femur and proximal tibia fracture (Figure 2). One of the patients sustained concomitant fractures that included an intra-articular distal femur fracture and extra-articular distal tibia fracture. Eighty percent of the patients showed radiological evidence of osteoarthritis and a history of knee pain prior to trauma. The mean time from admission to surgery was 9.3 ± 3.5 days. The mean operating time for the procedure was 140.4 ± 36.8 minutes. All surgeries used a medial parapatellar approach and no patella resurfacing was done. In all patients, a fully cemented Legacy constrained condylar total knee arthroplasty implant with tibia stem and/or femoral stem (Zimmer, Warsaw, Indiana) was used. Five of the patients were allowed full weight bearing on the second day after surgery. Four patients had restricted weight bearing due to underlying comorbidities and the complexity of the fractures (Table 1). One patient had to be on crutches due to a concomitant distal tibia fracture. The mean length of the hospital stay was 14 ± 7 days.

### Outcomes

The mean follow-up duration for the cohort was 22.3 ± 13.9 days with a mean knee score of 87.7 ± 10.0 and a mean functional score of 56.0 ± 41.9. Mean knee flexion was 96.0 ± 21.2°. No patients presented with fixed flexion deformity, extension lag or hyperextension of the involved leg. A lower functional knee score can be noted in patients who suffered from complications post-operatively; however, the differences were not statistically significant in this study. No revision surgery has been done, thus far during follow-up. There was no mortality noted in this cohort. A lower functional knee score can be noted in patients who suffered from complications post-operatively. Two patients were found to have surgical site infections (SSI) of the knee. These patients underwent a debridement and implant retention (DAIR) procedure with prolonged antibiotic therapy. One patient’s case was complicated by SSI of the distal tibia, which was managed with debridement and antibiotic therapy. Another patient, who did not have any post-surgical complications, had a low functional knee score due to a fixed flexion deformity of the contralateral knee, making ambulation and stair climbing difficult.

## Discussion

This study shows that early primary total knee replacement is a viable option for the treatment of acute knee fractures in the elderly population. The treatment outcomes at the early- and medium-term follow-up of this study, with a mean knee score of 87.7 ± 10.0 and a mean function knee score of 56.0 ± 41.9, are comparable to other studies (4–7, 9–23). Patients who were allowed early mobilisation after surgery showed the best results in this study. Pearse et al. (20) compared elderly patients who underwent total knee arthroplasty and ORIF for supracondylar femur fractures and found that total knee arthroplasty provided better walking ability and range of motion. A more recent study by Ebied et al. (22) with a total of 27 patients showed similarly good results. However, in their study, they splinted the patients’ knees for 4 weeks to allow for the reduction of soft tissue swelling and inflammation before proceeding to a knee replacement.

The implants used in primary total knee replacements after trauma include semi-constraint implants, hinge knees and megaprotheses (13, 17, 19). In this study, a constrained posterior stabilised implant was chosen due to osteoporosis and comminution of the fractures, causing balancing difficulties. At the same time, this implant enabled a more proximal or distal distribution of load to enable immediate weight bearing. The stem was not cemented to avoid difficulties during possible revision as suggested by Vermeire et al. (7). Any bone defect was filled with bone cement to provide better stability and enable weight bearing (10). However, Schwarz et al. (12), who treated simple split depression tibia fractures with standard arthroplasty implants, obtained comparable results.

A mortality rate of 22% was found in a mixed treatment mode and a 30% mortality rate was found in a study assessing the treatment of distal femur fractures with ORIF in elderly patients (24). Poor outcomes in our series were associated with post-operative complications, even though two out of four patients with bad functional outcomes arguably did not have complications related to the arthroplasty surgery. However, the statistical analysis of the relation between age, gender, duration of operation, presence of post-operative complication and outcome measures did not reveal any statistical significance, likely due to the small sample size. One patient had a knee score of 72 but a functional knee score of 5 due to surgical site infection of the ipsilateral distal tibia, disenabling the patient from bearing weight. One patient had a knee score of 70 and a painless knee range of motion of 0°–90° but could not ambulate well due to a contralateral knee fixed flexion deformity of 30° as well as knee pain due to osteoarthritis. Two patients who had developed surgical site infections of the knees and underwent the DAIR procedure had poor knee scores on follow-up.

Previous studies by Boureau et al. (13) and Parratte et al. (15) have shown that the revision rate for patients who underwent primary total knee replacement for acute fractures around the knee ranges from 9.5% to 11.5%; they agreed that this was less than the revision rate associated with osteosynthesis. This complication rate is lower than the complication rate in total knee replacements in post-traumatic osteoarthritis (21, 25, 26). Scott et al. (21) reported an early wound complication rate of 16% in patients who underwent total knee replacement after post-traumatic osteoarthritis due to a tibial plateau fracture. No mortality was noted during follow-up. The pre-existing medical comorbidities of this population greatly influence the outcome of early total knee arthroplasty in trauma. Appleton et al. (19) reported a mortality rate of 42% during the first year. Boureau et al. (13) reported a 14% mortality rate at the one-year follow-up. However, 62% of the studied population had American Society of Anaesthesiologist (ASA) classification scores of 3, which could explain the high mortality rates noted in the study.

Such extreme surgeries are not well practised by surgeons worldwide, mainly due to the complexity of the surgery itself, the risk of periprosthetic joint infection and the lack of long-term follow-up information. However, long-term follow-up after surgery on elderly patients is challenging as this group of patients has multiple comorbidities that potentially could contribute to early mortality. The main advantage of the surgery is that it can provide good results at the very beginning of the post-operative period, enabling immediate weight bearing, which in turn ensures better autonomy for the elderly patient.

## Conclusion

The main advantage of early total knee replacement in acute trauma of the knee in the elderly population lies in the rapid return to pre-fracture conditions and early weight bearing. We found that early total knee replacement helped our patients by hastening the recovery process. However, the indication to perform this surgery must be precise, and extensive planning is needed to minimize intra-operative complications. This study has demonstrated excellent short-term outcomes, which are comparable to multiple series published before this, but the surgery is not without the potential risk of infection (20% in our study) and poor functional outcome post-operatively. However, there was no statistical significance in the correlation between demographic characteristics and the presence of post-operative complications with the outcomes for patients. Thus, all variables were deemed to be confounders with the outcome of the surgery. Further research is needed to better understand the long-term outcomes of this surgery. The limitations of this study include the lack of a control group as well as the small study population.

## Figures and Tables

**Table 1:** Patients who underwent early total knee replacement due to fractures around the knee between January 2017 and December 2019

ID	Age	Gender	Femur #	Tibia #	Days to Op (days)	Duration of Op	Follow-up duration (months)	ROM on last follow-up, degrees (°)	Complications	Knee score	Functional score
A	64	M	33C1.1		10	3 h 7 min	47	0°–120°	-	97	100
B	63	M	33C2.1	43B1.1	14	2 h 24 min	12	0°–90°	-	98	90
C	67	F		41C3.3	6	1 h 39 min	42	0°–90°	-	97	80
D	58	M		41C3.1	15	1 h 52 min	28	0°–130°	-	88	90
E	72	F	33B2.3		9	1 h 37 min	23	0°–80°	-	93	100
F	65	F	33C2.2		7	1 h 50 min	24	0°–120°	-	92	50
G	55	F	33C2.2	41C3.3	12	2 h 47 min	10	0°–90°	SSI	85	45
H	64	M	33C1.1		9	3 h 20 min	23	0°–60°	SSI	85	0
I	60	M	33C2.1		7	2 h 10 min	7	0°–90°	SSI of distal tibia	72	5
J	76	F	33C1.1		4	2 h 38 min	7	0°–90°	-	70	0
